# Molecular mechanisms and clinical relevance of endothelial cell cross-talk in clear cell renal cell carcinoma

**DOI:** 10.48101/ujms.v129.10632

**Published:** 2024-05-08

**Authors:** Elin Sjöberg

**Affiliations:** Department of Immunology, Genetics and Pathology, Uppsala University, Uppsala, Sweden

**Keywords:** Kidney cancer, clear cell renal cell carcinoma, tumor microenvironment, vasculature, endothelial cells, immune cells

## Abstract

**Background:**

Clear cell renal cell carcinoma (ccRCC) is the most common renal cancer in adults and stands out as one of the most vascularized and immune-infiltrated solid tumors. Overproduction of vascular endothelial growth factor A promotes uncontrolled growth of abnormal vessels and immunosuppression, and the tumor microenvironment (TME) has a prominent role in disease progression, drug targeting and drug response, and for patient outcome.

**Methods:**

Studies of experimental models, large-scale omics approaches, and patient prognosis and therapy prediction, using gene expression signatures and tissue biomarker analysis, have been reviewed for enhanced understanding of the endothelium in ccRCC and the interplay with the surrounding TME.

**Results:**

Preclinical and clinical studies have discovered molecular mechanisms of endothelial cross-talk of relevance for disease progression, patient prognosis, and therapy prediction. There is, however, a lack of representative ccRCC experimental models. Omics approaches have identified clinically relevant subsets of angiogenic and immune-infiltrated tumors with distinct molecular signatures and distinct endothelial cell and immune cell populations in patients.

**Conclusions:**

Recent genetically engineered ccRCC mouse models together with emerging evidence from single cell RNA sequencing data open up for future validation studies, including multiplex imaging of ccRCC patient cohorts. These studies are of importance for therapy benefit and personalized treatment of ccRCC patients.

## Introduction

Renal cell carcinoma (RCC) is the most prevalent kidney cancer. One-third of patients manifests metastasis at diagnosis, and a similar fraction relapse after intended curative surgery (1). Among the three major histological subtypes, clear cell renal cell carcinoma (ccRCC), papillary RCC, and chromophobe RCC, ccRCC is the most common, with 80% of diagnosed patients. It is a peculiar tumor with high metabolic rate, augmented vascularization and immune infiltration, and unlike most cancers, patients do not respond to conventional chemo- and radiotherapy (1–4). The tumor microenvironment (TME) has been shown to play a crucial role in disease progression, patient survival, and therapeutic efficiency (5), and the immune microenvironment has surprisingly been linked to poor patient prognosis (6). The pathogenesis of ccRCC is characterized by an early genetic loss of von Hippel-Lindau (*VHL*), resulting in accumulation of HIF1α and HIF2α and elevated expression of target genes, including vascular endothelial growth factor A (VEGFA) (7, 8). Increased VEGFA production promotes a hyper-angiogenic state, with tortuous and hyperpermeable vessels, affecting immune cell infiltration, metastatic spread, and drug delivery (7, 9).

In the last decade, antiangiogenic therapy against VEGF/VEGFR-signaling, including tyrosine kinase inhibitors (TKIs), together with immune checkpoint blockade (ICB) have improved the survival of patients with advanced RCC, and combination therapies are now considered the backbone for systemic therapy (10–12). This has led to a paradigm shift for the treatment of metastatic disease. Still, not all patients benefit and some even progress. To individualize treatment regimen, it is critical to identify why certain patients respond. This review will give an overview of the tumor vasculature in ccRCC, and its relevance for disease progression, treatment response, and patient outcome. Examples of the interplay between the endothelial cells (ECs) and the surrounding tumor cells and immune cells will be given from preclinical studies and tissue biomarker studies. In addition, RCC EC-phenotypes and their clinical relevance, gained from recent omics-analysis, will be summarized.

## Molecular insight of EC-interactions from preclinical models

The vasculature is distinct between renal compartments and includes glomerular and cortical peritubular capillaries, and vasa recta bundles (13). Glomerular capillaries ensure serum filtration in Bowman’s capsule and further extend into peritubular capillaries in the renal cortex. The descending vasa recta (DVR) ensure blood flow from the cortex to the renal medulla, and the fenestrated ascending vasa recta (AVR) originate from capillaries in the medulla and transport blood back to the cortex, simultaneously ensuring reabsorption (14). The development and maintenance of the normal kidney vasculature is dependent on VEGFA/VEGFR2-signaling (13). However, sustained exposure in ccRCC generates dysfunctional vessels with loss of barrier integrity, which alters immune cell trafficking, promotes metastasis, and prevents drug delivery (7). There is a mutual relationship between tumor vascular permeability and immune cell suppression in cancer (9, 15), and hypoxia hampers antitumor immunity by the expansion of regulatory T-cell (Tregs), myeloid-derived suppressor cells (MDSCs), and tumor-associated macrophages (TAMs) (15, 16). Moreover, chronic antigen exposure will result in an exhausted state of intratumoral T-cells (17, 18). In addition to the immunosuppressive effects of hypoxia, VEGF-signaling can also suppress immune functions (19–21) of importance for cancer patient survival (22). Until recently, the lack of representative immune competent preclinical tumor models has brought challenges for the exploration of the cellular and molecular cross-talk in ccRCC (23). Patient-derived xenografts have given important insight of the interplay in the TME (24). Notably, genetically engineered mouse models are now providing new opportunities, although not fully able to mimic human disease (25–27).

### Cellular interplay affecting tumor progression, immune suppression, and vascular normalization

In our recent publication, we identified a PLCγ/eNOS/Src-pathway downstream of the tyrosine phosphorylation site pY1173/Y1175 (mouse/human) in VEGFR2, of clinical relevance in ccRCC (28). Mechanistically, phospholipase Cγ (PLCγ)-induced protein kinase C (PKC) and Ca^2+^ activated endothelial nitric oxide synthase (eNOS), followed by enhanced nitration and activation of Src and augmented vascular permeability by VE-cadherin turn-over (28). Pathway inhibition in subcutaneous tumors using mice with an inactivating Y1173F mutation (*Vegfr2*^Y1173F/+^) led to vascular normalization, and as a result, tumors responded better to chemo- and immunotherapy. Furthermore, the presence of immunosuppressive cytokines, Tregs, and B-cells was reduced *in vivo* and correlated to PLCγ expression in ccRCC patients. These data demonstrate a paracrine cross-talk involving VEGFA secretion by tumor cells, which activate PLCγ signaling in the endothelium to reduce antitumor immunity (28).

Evidence from a mouse model with *Vhlh* (mouse *VHL* allele)-deficient renal tubular cells (*Hoxb7-Cre-GFP;Vhlh^fl/fl^*) delineated a paracrine signaling pathway of Oncostatin M (OSM), produced by VHL-deficient cancer cells interacting with its receptor (OSMR) expressed on ECs. Activation altered the endothelial gene expression profile, with upregulation of inflammatory cytokines (*Il-6*), chemokines (*Ccl2, Ccl7*), and adhesion markers (*Sele*, *Selp*, *Vcam1*, and *Icam1*). This was followed by TAM polarization (i.e. polarization to a tumor promoting phenotype) and diminished barrier function due to ECs undergoing endothelial-to-mesenchymal transition (EndMT), followed by increased vascular permeability, tumor cell extravasation, and metastasis formation. Double knock down of *Vhlh* and *Osmr* or treatment with an OSM inhibitory antibody *in vivo* rescued the pro-tumoral phenotype (29). Of note, the most upregulated gene in ECs was Arginase 1 known to modulate eNOS activity, and in line with our recent results (28), this highlights the importance of eNOS in the regulation of the vascular barrier in RCC. Tumor immune suppression in RCC was further explored in a study where cross-talk between cells was investigated by spatial localization. Analysis of 12 human RCC patients revealed an EMT profile of cancer cells at the normal-tumor border that co-localized with a subset of tissue resident *IL1B*-expressing macrophages (30). *IL1B*-expressing macrophages were previously shown to promote an immunosuppressive micro-milieu and tumor growth in the syngeneic RENCA mouse model, when the murine RENCA RCC cells were injected subcutaneously in Balb/c mice. Combination therapy against IL1β and anti-PD-1 or the TKI cabozantinib promoted tumor regression as compared to monotherapy and specifically decreased the presence of immunosuppressive macrophages and MDSCs (31).

Other angiogenic factors, in addition to VEGF, were also shown to play a role in RCC vascular remodeling and progression *in vivo*, demonstrated by EC-specific removal of the GPCR-coupled kinase PI3Kβ in *Pik3cb*^fl/fl^;*Tie2-CreERT2* mice (32). Mice with EC-specific PI3Kβ knock down that were implanted with Lewis lung carcinoma (LLC1) or B16F10 melanoma tumors displayed reduced tumor growth and lung metastasis, and enhanced vascular normalization and better response to the antiangiogenic TKI sunitinib (32).

Direct interactions of ECs and cancer cells by complex formation between VEGFR2 and the co-receptor Neuropillin 1 (NRP1) were demonstrated to reduce tumor formation in experimental models and were of clinical relevance for ccRCC and pancreatic adenocarcinoma (PDAC) (33–35). The expression of NRP1 on perivascular cancer cells led to VEGFR2 binding in a trans configuration, delayed EC-signaling including PLCγ and ERK, and repressed tumor initiation, angiogenesis, vessel branching, and tumor cell proliferation *in vivo* (33, 34). In a publication by Cao et al., the closely related NRP2 expressed by RCC tumor cells was shown to interact with α5 integrin expressed on ECs to promote vascular adhesion, extravasation, and metastasis in experimental tumors *in vivo*. NRP2 expression was also enhanced in metastatic ccRCC patients and correlated to advanced tumor stage and worse outcome (36).

In addition to affect immune cell infiltration and activation, antiangiogenic VEGF-targeting has also been shown to differentially affect primary- and metastatic RCC tumors *in vivo*. Tumor vascularization occurs not only via angiogenesis (37) but also via hijacking of preexisting vessels by co-option (38, 39). Insights from the immunocompetent RENCA model showed that primary implanted tumors were sensitive to sunitinib treatment. However, the formation of lung metastasis was dependent on vessel co-option, and consequently, a reduced sunitinib sensitivity was recorded (40). Single cell RNA sequencing (scRNA-seq) demonstrated that ECs in co-opted vessels were transcriptionally similar to quiescent healthy ECs, which may complicate vascular targeting. It is possible that co-option also could explain why certain metastatic ccRCC patients do not respond to antiangiogenic therapy (40).

## Characterization of the immune microenvironment in ccRCC

Single cell analysis has recently shed light on proximal tubular cells as the cell of origin for ccRCC (41–43). In addition to the well characterized malignant cells, the immune compartment has been comprehensively characterized, and immunosuppressive and exhausted cells were enriched in patients with advanced disease and in ICB responders (18, 30, 44–46). Bi et al. showed that immune checkpoint inhibition remodeled the TME and augmented an exhausted T-cell population, and unexpectedly, both a pro-inflammatory and immunosuppressive phenotype of TAMs, suggesting immune system adaptation, which might result in treatment resistance (45). ScRNA-seq has further been utilized to generate cell population-signatures of predictive value across cohorts. Krishna et al. performed scRNA-seq of two untreated and four ICB treated patients and revealed the presence of tissue resident CD8^+^ T-cells that expanded upon treatment and predicted the therapeutic benefit (46). Analysis of the resident CD8^+^ T-cell-signature in bulk RNA-seq data from the JAVELIN 101 cohort (10) revealed enhanced progression-free survival (PFS) in patients treated with avelumab (anti-PDL1) + axitinib (anti-VEGF), suggesting that patients with a high number of these resident T-cells will respond better to ICB (46). In concordance with the T-cells identified in Bi et al., these cells also expressed the inhibitory immune checkpoint proteins PD-1 and LAG3 and effector molecules IFNγ and PRF1 (45, 46). These studies support the presence of a dysfunctional immune system in ccRCC, which negatively impacts on patient survival but does predict which patients will benefit from immune checkpoint inhibition.

In addition to VEGF secretion by ccRCC cells, which activates the receptors expressed on tumor ECs, Young and colleagues identified macrophages as an additional source of VEGF production (41). The relevance of macrophages was reported by Zhang et al., who defined two macrophage clusters with opposing effects on patient outcome in the ccRCC publicly available TCGA data set (KIRC) (42). Several publications have identified a subpopulation of TAMs in ccRCC patients expressing *TREM2* (30, 43, 44, 47), associated with poor patient prognosis (43) and elevated expression of immune checkpoint ligands as well as VEGFA, suggesting important functions for immune suppression and targeting (47). Notably, anti-PD-1 therapy has shown better efficacy in *Trem2*^-/-^ mice or in combination with anti-TREM2 blockade (48).

Cancer cell-induced T-cell exhaustion is a mechanism for immune evasion of tumors (17). The identification of clonally expanded T-cells both within solid tumors and patient-matched peripheral blood in multiple cancers suggested that exhausted T-cells get replaced to overcome immune evasion, in particularly in patients who respond to ICB (49). The spatial heterogeneity of ccRCC tumor cells and the TME were addressed by Li et al., and multi-region-based genomic- and scRNA-seq were performed in 12 patients (30). Analysis of paired blood samples showed that exhausted tissue resident CD8^+^ T-cells appeared unable to recirculate in the blood stream and remained trapped within tumors (30) in accordance with a previous report on advanced ccRCC (50).

B-cells in ccRCC have been sparsely studied, which might be reflected by low infiltration (44). However, a minority group of patients with high intratumoral B-cells was shown to display poor prognosis (51). If these B-cells exhibit an immunosuppressive or dysfunctional phenotype has not yet been outlined. In addition, a multi-omics approach has also revealed the importance of B-cells for ICB response in RCC. B-cells were more present in responders as compared to non-responders, and notably, responders had higher frequencies of memory B-cells and non-responders of naïve B-cells (52).

## Characterization of the human ccRCC TME has identified clinically relevant tumor subsets with distinct molecular signatures

Two molecular subtypes of ccRCC were previously identified by a 34-gene expression signature: one characterized by elevated angiogenesis and metabolism, and improved patient outcome, and the other one by worse survival and immunosuppression, and wound healing (53, 54). Additional reports have confirmed molecular subtypes with a more aggressive disease course characterized by high immune cell-infiltration and dysfunctional immune cells, as compared to highly vascularized tumors (4, 55–57). Four subtypes of ccRCC (ccrcc1-4) with distinct treatment responses to sunitinib have also been reported. The ccrcc4 subtype was characterized by the expression of immunosuppressive Treg markers Foxp3, IL-10, and TGFβ and the checkpoint receptors PD-1 and LAG-3, as well as enriched B-cell and T-cell transcripts. Out of 98 patients analyzed, the ccrcc4 subgroup displayed the highest fraction (27%) of non-responders and shortest recurrence rate and PFS (56). Clark and colleagues (58) performed multi-omics characterization of 103 ccRCC patients and paired normal adjacent tissue. Four subtypes were identified: CD8^+^ inflamed (1), CD8^-^ inflamed (2), VEGF immune desert (3), and metabolic immune desert (4) with distinct TME signatures. The CD8^+^ inflamed subtype had elevated levels of CTLA4, PD-1, PD-L1, and PD-L2 and INFγ signaling. The CD8^-^ inflamed subtype expressed innate immune genes and enrichment of fibroblasts and ECs. Similarly, angiogenesis was also enriched in the VEGF immune desert-subtype (i.e. tumors devoid of immune cells) with enhanced Wnt/β-catenin, RAP1, and Notch-signaling, suggesting distinct tumor vascular beds between these two subtypes. The metabolic immune desert tumors were characterized by low infiltration of immune cells and enhanced metabolic pathways, mTOR-signaling, and a *MYC* target gene signature. A survival benefit was demonstrated for the VEGF immune desert subtype, while CD8^+^ inflamed patients had a poor prognosis, in line with previous studies (4, 55–57). Findings also suggested that the VEGF immune desert- and CD8^+^-inflamed subtypes would be responsive to antiangiogenic therapy and ICB, respectively (58). In concordance, Motzer et al. identified seven molecular subsets of advanced RCC by unsupervised transcriptomics of 823 patients (59). Clusters 1 (angiogenic/stromal) and 2 (angiogenic) showed high angiogenesis, and cluster 4 (T-effector/proliferative) high T-cell activity. Treatment with atezolizumab + bevacizumab versus sunitinib demonstrated an ICB-benefit in cluster 4-patients, showing immune-enrichment and poor-angiogenesis. Patients in the high-angiogenesis clusters 1 and 2 had longer PFS in both treatment arms, most likely due to the presence of antiangiogenic therapy (59). In line with previous findings, this confirms the prominence of adaptive immunity for the benefit of immune checkpoint inhibition and high vascularization for responsiveness to VEGF/VEGFR2 blockade in advanced RCC.

## Clinical relevance of ccRCC endothelial subsets identified by scRNA-seq

As compared to the immune microenvironment, less attention has been directed toward the ECs in human ccRCC. However, tumor-enriched EC-populations have been described in recent studies, especially a subpopulation of ECs expressing the chemokine receptor gene *ACKR1* (ACKR1^+^), linked to shorter disease-free survival (DFS) and overall survival (OS) (30, 41–43, 47). Interestingly, the ACKR1^+^ ECs appear distinct from ECs expressing VEGFR2 (41–43), implicating the presence of EC-populations with different treatment responses and clinical significance. Additionally, Li et al. examined the spatial localization and found ACKR1^+^ ECs enriched in the tumor core as compared to the tumor-normal interface of ccRCC (30).

ScRNA-seq of tumor tissue and adjacent normal tissue from 9 ccRCC treatment-naïve patient identified high transcriptional remodeling and immunosuppression in the TME, as compared to normal tissue (43). Five EC clusters were identified (Endo-1 to -5), and two were enriched in the tumor compartment (Endo-1 and -2) as compared to normal kidney. Endo-1 was the predominant cell cluster, expressed a signature of *PVLAP, CA2, PARC, INSR*, and *IGFBP7*, and enhanced VEGFR2 (*KDR*) expression. The Endo-2 cluster expressed the vascular genes *ACKR1, VCAM1*, and *VWF* and specifically venous EC genes (*GPM6A*, *CYP1B1*, and *MMRN1*). Hu et al. identified six clusters of ECs, of which two were enriched in ccRCC as compared to benign kidney. These two clusters expressed *KCNE3* (KCNE3^+^ cluster 1) and *ACKR1* (ACKR1^+^ cluster 6), and the latter was associated with decreased patient survival in the TCGA KIRC cohort (47), demonstrating clinical impact of a specific EC-subpopulation in ccRCC. Furthermore, 14 genes were enriched in the tumor endothelium irrespectively of subclusters, including *VWF, ENPP2, IGFBP3*, and *CAV1*, and authors also validated the presence of VWF^+^ENPP2^+^ expressing ECs in ccRCC by immunostaining (47). Two major EC-populations were identified in ccRCC tissue by Zhang and colleagues, as compared to five clusters in benign renal tissue (42). The major EC cluster, ccRCC-AVR-1 originated from AVR, was positive for *PLVAP* and had upregulated endothelin receptor type B (*EDNRB*), von Willebrand factor (*VWF*), and heparan sulfate proteoglycan 2 (*HSPG2*). This cluster exhibited elevated expression of VEGFR-genes as compared to the minor ccRCC-AVR-2 cluster that instead expressed *ACKR1* and *SELP*, suggesting that patients with high number of AVR-2 vessels might not benefit from antiangiogenic treatment (42). To understand the interplay of TME cell types of importance for ccRCC progression, authors mapped receptor–ligand interactions and identified OSM/OSMR signaling between ccRCC cells and macrophages (42), in agreement with preclinical evidence (29). In addition, endothelin ligand 1 (EDN1) expressed on malignant cells interacted with the EDNRB receptor, previously identified as an independent prognostic marker in ccRCC (60), and expressed in the tumor endothelium. An additional scRNA-seq study exploring the spatial transcriptome of ccRCC identified two major EC-subsets, *IGFBP3*^+^ ECs and collagen ECs enriched in tumor tissue, as compared to adjacent normal tissue. The *IGFBP3*^+^ ECs, which have been described in additional studies (43, 47), were mainly present in the tumor core, while the collagen ECs were more prominent in the tumor-normal interface (30).

## Tissue biomarker assessment of prognosis and therapy prediction in RCC

### The vasculature as a prognostic or predictive marker in RCC

The clinical relevance of RCC vascularization has been disputed. Two distinct types of blood vessels with contrasting clinical impact were described in 2007 by Yao et al. Undifferentiated CD31^+^/CD34^-^ and differentiated CD34^+^ vessels correlated to decreased and improved ccRCC survival, respectively (61), indicating ECs heterogeneity and suggesting relevant selection of vessel markers for the evaluation of disease course and therapy prediction. Recent studies have correlated microvessel density (MVD) to a favorable prognosis but were unable to show predictive benefit (62, 63). MVD of 822 high-risk RCC patients, initially enrolled in the ECOG-ACRIN 2805 phase III trial comparing adjuvant sunitinib, sorafenib, or placebo (64), was analyzed by CD34 immunostaining. In the entire cohort, MVD was revealed as an independent positive prognostic factor for OS (63). However, the survival benefit was reduced in patients receiving adjuvant sunitinib or sorafenib, as compared to placebo, proposing MVD as a purely prognostic biomarker and not predictive for therapeutic benefit (63). In a study by Denize et al., vessel density was explored in metastatic RCC patients treated with the TKI cabozantinib versus the mTOR inhibitor everolimus. High CD31^+^ MVD was positively correlated to PFS both in uni-variable and multivariable analysis but did not predict benefit of cabozantinib (62). In disagreement with these studies, Motzer et al. showed that two angiogenic signatures (12, 65) enhanced PFS in 886 advanced RCC patients treated with the multi-target TKI sunitinib versus axitinib (against VEGFRs) + avelumab (anti-PD-L1). This suggests a predictive value of tumor vascularization for sunitinib therapy. On the contrary, immune signatures were only predictive for the combination therapy (65). This is in line with previous findings in advanced ccRCC patients treated with atezolizumab + bevacizumab versus sunitinib, where a T-cell signature was associated with enhanced PFS in the combination arm, suggesting that atezolizumab + bevacizumab is beneficial for patients who already exhibit antitumor immunity (12).

### Endothelial tissue biomarkers for RCC prognosis or therapy prediction

The involvement of the tumor endothelium in RCC progression and patient survival has been investigated in a number of studies ranging from the multi-omics-approaches, as described above, to single biomarker-studies. EC-cross-talk, identified by tissue analysis with an impact on prognosis and/or treatment prediction, will be discussed here.

#### Prognostic tumor endothelial biomarkers

A 16-gene signature was previously shown to predict shorter disease-specific survival (DSS), DFS, and OS in ccRCC (55). Of note, four genes were vascular markers, *NOS3, APOLD1, EDRNB*, and *PPAP2B*, and even though the signature predicted high risk of recurrence, the vascular genes were associated with low recurrence risk, supporting previous findings that the tumor vasculature in ccRCC correlates to improved patient outcome (55). On the contrary, EC-signaling in RCC correlating to worse patient outcome has also been identified (28, 66). A clinically relevant paracrine cross-talk between ccRCC tumor cells and ECs involving a VEGFR2 pY1175/PLCγ signaling pathway was recently shown to contribute to abnormal vessel functions *in vivo* and impact on disease course (28). PLCγ was predominantly enriched in the tumor endothelium and was further shown to be an independent biomarker for DSS in ccRCC (28). Paracrine cross-talk between tumor cells in ECs was also shown by Wragg et al., who reported elevated levels of melanoma cell adhesion molecule (MCAM) expression induced by VEGFA in ccRCC endothelium, as compared to normal kidney. In two RCC cohorts, endothelial MCAM expression correlated to advanced disease stage and poor prognosis (66). Direct cell-to-cell interactions in the TME have also shown to be of clinical importance (34–36). The formation of NRP1/VEGFR2 trans complexes identified by proximity ligation assay, or the presence of perivascular NRP1, was identified to correlate to a beneficial RCC prognosis in three independent cohorts (35), in line with previously shown for pancreatic cancer (34). Perivascular NRP1 showed a survival advantage both in a 314-treatment naïve patient cohort and in a 64-patient cohort treated with sunitinib after surgical resection, which suggests NRP1/VEGFR2 signaling to be purely prognostic and not predictive for sunitinib treatment.

#### Predictive tumor endothelial biomarkers

Epidermal growth factor latrophilin and seven transmembrane domain-containing protein 1 (ELTD1), initially identified as a good prognostic biomarker in ccRCC (67), was further shown to predict sunitinib response (68). In a tumor tissue microarray (TMA) consisting of 99 sunitinib-treated patients with advanced RCC, ELTD1 expression was confined to the endothelium and associated with enhanced PFS. No survival benefit could be shown for sorafenib, and thus, ELTD1 was concluded to be a response-predictive marker solely for sunitinib treatment (68). In an additional report, the ligand for the receptor tyrosine kinase Tie2, Angiopoietin-2 (Ang-2), predicted sunitinib response in metastatic RCC patients. Results showed a selective and variable Ang-2 expression in the tumor endothelium, correlating to enhanced vascular density and initial clinical benefit to sunitinib but no effect on patient outcome (69). In addition, analysis of a small cohort of 15 advanced RCC patients treated with Nivolumab identified the immune-inhibitory Indoleamine 2,3-dioxygenase 1 (IDO-1) as a predictive EC-biomarker. IDO-1 was predominantly expressed in the tumor vasculature, more frequently in Nivolumab responders as compared to non-responders, and correlated to improved PFS during immunotherapy treatment and inversely correlated to CD4/CD8 ratio (70).

## Conclusions

**Figure 1:**
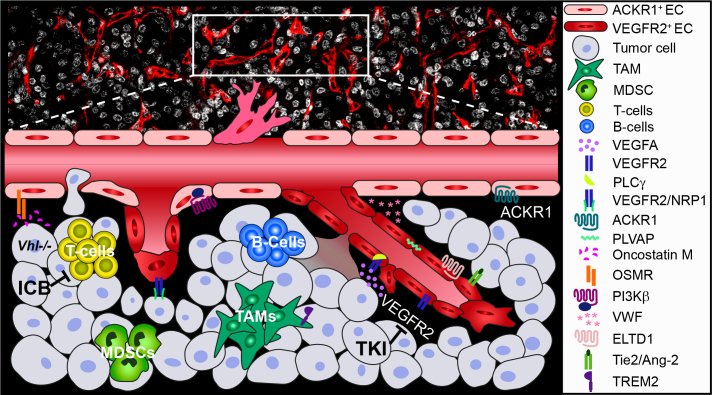
Tumor microenvironmental (TME) interplay in ccRCC; insights from preclinical and clinical research. CcRCC tumors are highly vascularized and infiltrated with immune cells. Endothelial cell (EC)-subsets have been identified, including ACKR1^+^ ECs distinct from VEGFR2^+^ ECs. The different ECs not only express unique markers, including PLVAP, but also share expression, including vWF. Paracrine signaling involves elevated VEGFA from tumor cells that activates PLCγ in ECs and enhances tumor vascular leakage and immunosuppression, important for patient survival. VEGFA also modulates the immune microenvironment by infiltration of exhausted T-cells, MDSCs, and tumor-associated macrophages (TAMs). TREM2^+^ TAMs promote tumor progression and worse outcome. Another example of paracrine cross-talk is oncostatin M secretion by *Vhl*-deficient cells, which binds EC OSMR to enhance metastatic spread in mice. The G-protein coupled receptor (GPCR) ELTD1 and the angiopoietin-2 ligand for the receptor tyrosine kinase Tie2 have been identified as predictive markers for sunitinib response in RCC patients, while the GPCR-coupled kinase PI3Kβ promotes sunitinib resistance and vascular permeability in experimental mouse models. Direct interactions of tumor cells and ECs involve the VEGFR2 co-receptor NRP1, which suppresses angiogenesis and tumor cell proliferation and promotes RCC patient survival. Systemic therapy for advanced ccRCC patients targets the TME and includes immune checkpoint blockade (ICB) in combination with antiangiogenic tyrosine kinase inhibitors (TKIs).

Multi-omics of human patients together with data from experimental mouse models highlight the molecular interplay between tumor cells and the TME in kidney cancer. Findings have revealed distinct ccRCC EC-populations with expression of unique markers and important mechanistic characteristics, of which examples are provided in Figure 1. We are in the beginning of an era of in-depth large-scale characterization of the ccRCC endothelial landscape and additional stromal cells. A limitation of published scRNA-seq reports is the number of patients analyzed and validation of identified subclusters. More extensive studies are encouraged to overcome inter-patient heterogeneity, together with validation of protein expression and clinical relevance in larger well-annotated tumor collections. Furthermore, the inclusion of patients from the same histological RCC subtype and treatment regimen is of importance. Current studies have often not distinguished between the type of ICB treatment or if patients also received TKI-treatment, which may have influenced the conclusions. The development of improved immune competent genetically engineered mouse models for mechanistic insight, and multiplex imaging to understand the cellular and molecular interplay on a spatial level is also needed to delineate the functional relationship between the tumor endothelium and immune compartment in ccRCC. An enhanced understanding of the ccRCC TME further generates possibilities for the identification of drug targets and predictive biomarkers, to better personalize treatment of patients.

## Disclosure statement

The author reports no conflict of interest.

## Funding

This study was funded by The Swedish Society for Medical Research (SSMF) (P17-0144), Magnus Bergvalls stiftelse (2021-04476, 2022-388, and 2023-719), and Åke Wibergs stiftelse (M22-0141 and M23-0182).

## References

[1] Choueiri TK, Motzer RJ. Systemic therapy for metastatic renal-cell carcinoma. N Engl J Med. 2017;376:354–66. doi: 10.1056/NEJMra1601333 28121507

[2] Rooney MS, Shukla SA, Wu CJ, Getz G, Hacohen N. Molecular and genetic properties of tumors associated with local immune cytolytic activity. Cell. 2015;160:48–61. doi: 10.1016/j.cell.2014.12.033 25594174 PMC4856474

[3] Hakimi AA, Reznik E, Lee CH, Creighton CJ, Brannon AR, Luna A, et al. An integrated metabolic atlas of clear cell renal cell carcinoma. Cancer Cell. 2016;29:104–16. doi: 10.1016/j.ccell.2015.12.004 26766592 PMC4809063

[4] Senbabaoglu Y, Gejman RS, Winer AG, Liu M, Van Allen EM, de Velasco G, et al. Tumor immune microenvironment characterization in clear cell renal cell carcinoma identifies prognostic and immunotherapeutically relevant messenger RNA signatures. Genome Biol. 2016;17:231. doi: 10.1186/s13059-016-1092-z 27855702 PMC5114739

[5] Vuong L, Kotecha RR, Voss MH, Hakimi AA. Tumor microenvironment dynamics in clear-cell renal cell carcinoma. Cancer Discov. 2019;9:1349–57. doi: 10.1158/2159-8290.CD-19-0499 31527133 PMC6774890

[6] Fridman WH, Zitvogel L, Sautes-Fridman C, Kroemer G. The immune contexture in cancer prognosis and treatment. Nat Rev Clin Oncol. 2017;14:717–34. doi: 10.1038/nrclinonc.2017.101 28741618

[7] Choueiri TK, Kaelin WG, Jr. Targeting the HIF2-VEGF axis in renal cell carcinoma. Nat Med. 2020;26:1519–30. doi: 10.1038/s41591-020-1093-z 33020645

[8] George DJ, Kaelin WG, Jr. The von Hippel-Lindau protein, vascular endothelial growth factor, and kidney cancer. N Engl J Med. 2003;349:419–21. doi: 10.1056/NEJMp030061 12890838

[9] Jain RK. Antiangiogenesis strategies revisited: from starving tumors to alleviating hypoxia. Cancer Cell. 2014;26:605–22. doi: 10.1016/j.ccell.2014.10.006 25517747 PMC4269830

[10] Motzer RJ, Penkov K, Haanen J, Rini B, Albiges L, Campbell MT, et al. Avelumab plus axitinib versus sunitinib for advanced renal-cell carcinoma. N Engl J Med. 2019;380:1103–15. doi: 10.1056/NEJMoa1816047 30779531 PMC6716603

[11] Rini BI, Plimack ER, Stus V, Gafanov R, Hawkins R, Nosov D, et al. Pembrolizumab plus axitinib versus sunitinib for advanced renal-cell carcinoma. N Engl J Med. 2019;380:1116–27. doi: 10.1056/NEJMoa1816714 30779529

[12] McDermott DF, Huseni MA, Atkins MB, Motzer RJ, Rini BI, Escudier B, et al. Clinical activity and molecular correlates of response to atezolizumab alone or in combination with bevacizumab versus sunitinib in renal cell carcinoma. Nat Med. 2018;24:749–57. doi: 10.1038/s41591-018-0053-3 29867230 PMC6721896

[13] Augustin HG, Koh GY. Organotypic vasculature: from descriptive heterogeneity to functional pathophysiology. Science. 2017;357(6353):eaal2379. doi: 10.1126/science.aal2379 28775214

[14] Apelt K, Bijkerk R, Lebrin F, Rabelink TJ. Imaging the renal microcirculation in cell therapy. Cells. 2021;10:1087. doi: 10.3390/cells10051087 34063200 PMC8147454

[15] Huang Y, Kim BYS, Chan CK, Hahn SM, Weissman IL, Jiang W. Improving immune-vascular crosstalk for cancer immunotherapy. Nat Rev Immunol. 2018;18:195–203. doi: 10.1038/nri.2017.145 29332937 PMC5922422

[16] Qian BZ, Pollard JW. Macrophage diversity enhances tumor progression and metastasis. Cell. 2010;141:39–51. doi: 10.1016/j.cell.2010.03.014 20371344 PMC4994190

[17] Wherry EJ, Kurachi M. Molecular and cellular insights into T cell exhaustion. Nat Rev Immunol. 2015;15:486–99. doi: 10.1038/nri3862 26205583 PMC4889009

[18] Braun DA, Street K, Burke KP, Cookmeyer DL, Denize T, Pedersen CB, et al. Progressive immune dysfunction with advancing disease stage in renal cell carcinoma. Cancer Cell. 2021;39:632–48.e8. doi: 10.1016/j.ccell.2021.02.013 33711273 PMC8138872

[19] Ko JS, Zea AH, Rini BI, Ireland JL, Elson P, Cohen P, et al. Sunitinib mediates reversal of myeloid-derived suppressor cell accumulation in renal cell carcinoma patients. Clin Cancer Res. 2009;15:2148–57. doi: 10.1158/1078-0432.CCR-08-1332 19276286

[20] Finke JH, Rini B, Ireland J, Rayman P, Richmond A, Golshayan A, et al. Sunitinib reverses type-1 immune suppression and decreases T-regulatory cells in renal cell carcinoma patients. Clin Cancer Res. 2008;14:6674–82. doi: 10.1158/1078-0432.CCR-07-5212 18927310

[21] Gabrilovich DI, Chen HL, Girgis KR, Cunningham HT, Meny GM, Nadaf S, et al. Production of vascular endothelial growth factor by human tumors inhibits the functional maturation of dendritic cells. Nat Med. 1996;2:1096–103. doi: 10.1038/nm1096-1096 8837607

[22] Voron T, Colussi O, Marcheteau E, Pernot S, Nizard M, Pointet AL, et al. VEGF-A modulates expression of inhibitory checkpoints on CD8+ T cells in tumors. J Exp Med. 2015;212:139–48. doi: 10.1084/jem.20140559 25601652 PMC4322048

[23] Wolf MM, Kimryn Rathmell W, Beckermann KE. Modeling clear cell renal cell carcinoma and therapeutic implications. Oncogene. 2020;39:3413–26. doi: 10.1038/s41388-020-1234-3 32123314 PMC7194123

[24] Wang T, Lu R, Kapur P, Jaiswal BS, Hannan R, Zhang Z, et al. An empirical approach leveraging tumorgrafts to dissect the tumor microenvironment in renal cell carcinoma identifies missing link to prognostic inflammatory factors. Cancer Discov. 2018;8:1142–55. doi: 10.1158/2159-8290.CD-17-1246 29884728 PMC6125163

[25] Gu YF, Cohn S, Christie A, McKenzie T, Wolff N, Do QN, et al. Modeling renal cell carcinoma in mice: Bap1 and Pbrm1 inactivation drive tumor grade. Cancer Discov. 2017;7:900–17. doi: 10.1158/2159-8290.CD-17-0292 28473526 PMC5540776

[26] Bailey ST, Smith AM, Kardos J, Wobker SE, Wilson HL, Krishnan B, et al. MYC activation cooperates with Vhl and Ink4a/Arf loss to induce clear cell renal cell carcinoma. Nat Commun. 2017;8:15770. doi: 10.1038/ncomms15770 28593993 PMC5472759

[27] Harlander S, Schonenberger D, Toussaint NC, Prummer M, Catalano A, Brandt L, et al. Combined mutation in Vhl, Trp53 and Rb1 causes clear cell renal cell carcinoma in mice. Nat Med. 2017;23:869–77. doi: 10.1038/nm.4343 28553932 PMC5509015

[28] Sjoberg E, Melssen M, Richards M, Ding Y, Chanoca C, Chen D, et al. Endothelial VEGFR2-PLCgamma signaling regulates vascular permeability and anti-tumor immunity through eNOS/Src. J Clin Invest. 2023;133(20):e161366. doi: 10.1172/JCI161366 37651195 PMC10575733

[29] Nguyen-Tran HH, Nguyen TN, Chen CY, Hsu T. Endothelial reprogramming stimulated by oncostatin M promotes inflammation and tumorigenesis in VHL-deficient kidney tissue. Cancer Res. 2021;81:5060–73. doi: 10.1158/0008-5472.CAN-21-0345 34301760 PMC8974431

[30] Li R, Ferdinand JR, Loudon KW, Bowyer GS, Laidlaw S, Muyas F, et al. Mapping single-cell transcriptomes in the intra-tumoral and associated territories of kidney cancer. Cancer Cell. 2022;40:1583–99.e10. doi: 10.1016/j.ccell.2022.11.001 36423636 PMC9767677

[31] Aggen DH, Ager CR, Obradovic AZ, Chowdhury N, Ghasemzadeh A, Mao W, et al. Blocking IL1 beta promotes tumor regression and remodeling of the myeloid compartment in a renal cell carcinoma model: multidimensional analyses. Clin Cancer Res. 2021;27:608–21. doi: 10.1158/1078-0432.CCR-20-1610 33148676 PMC7980495

[32] Azad AK, Zhabyeyev P, Vanhaesebroeck B, Eitzen G, Oudit GY, Moore RB, et al. Inactivation of endothelial cell phosphoinositide 3-kinase beta inhibits tumor angiogenesis and tumor growth. Oncogene. 2020;39:6480–92. doi: 10.1038/s41388-020-01444-3 32879446

[33] Koch S, van Meeteren LA, Morin E, Testini C, Westrom S, Bjorkelund H, et al. NRP1 presented in trans to the endothelium arrests VEGFR2 endocytosis, preventing angiogenic signaling and tumor initiation. Dev Cell. 2014;28:633–46. doi: 10.1016/j.devcel.2014.02.010 24656741

[34] Morin E, Sjoberg E, Tjomsland V, Testini C, Lindskog C, Franklin O, et al. VEGF receptor-2/neuropilin 1 trans-complex formation between endothelial and tumor cells is an independent predictor of pancreatic cancer survival. J Pathol. 2018;246:311–22. doi: 10.1002/path.5141 30027561 PMC6221118

[35] Morin E, Lindskog C, Johansson M, Egevad L, Sandstrom P, Harmenberg U, et al. Perivascular Neuropilin-1 expression is an independent marker of improved survival in renal cell carcinoma. J Pathol. 2020;250:387–96. doi: 10.1002/path.5380 31880322 PMC7155095

[36] Cao Y, Hoeppner LH, Bach S, Guangqi E, Guo Y, Wang E, et al. Neuropilin-2 promotes extravasation and metastasis by interacting with endothelial alpha5 integrin. Cancer Res. 2013;73:4579–90. doi: 10.1158/0008-5472.CAN-13-0529 23689123 PMC3774599

[37] Folkman J. Tumor angiogenesis: therapeutic implications. N Engl J Med. 1971;285:1182–6. doi: 10.1056/NEJM197111182852108 4938153

[38] Kuczynski EA, Vermeulen PB, Pezzella F, Kerbel RS, Reynolds AR. Vessel co-option in cancer. Nat Rev Clin Oncol. 2019;16:469–93. doi: 10.1038/s41571-019-0181-9 30816337

[39] Qian CN. Hijacking the vasculature in ccRCC--co-option, remodelling and angiogenesis. Nat Rev Urol. 2013;10:300–4. doi: 10.1038/nrurol.2013.26 23459032

[40] Teuwen LA, De Rooij L, Cuypers A, Rohlenova K, Dumas SJ, Garcia-Caballero M, et al. Tumor vessel co-option probed by single-cell analysis. Cell Rep. 2021;35:109253. doi: 10.1016/j.celrep.2021.109253 34133923

[41] Young MD, Mitchell TJ, Vieira Braga FA, Tran MGB, Stewart BJ, Ferdinand JR, et al. Single-cell transcriptomes from human kidneys reveal the cellular identity of renal tumors. Science. 2018;361:594–9. doi: 10.1126/science.aat1699 30093597 PMC6104812

[42] Zhang Y, Narayanan SP, Mannan R, Raskind G, Wang X, Vats P, et al. Single-cell analyses of renal cell cancers reveal insights into tumor microenvironment, cell of origin, and therapy response. Proc Natl Acad Sci U S A. 2021;118(24):e2103240118. doi: 10.1073/pnas.2103240118 34099557 PMC8214680

[43] Alchahin AM, Mei S, Tsea I, Hirz T, Kfoury Y, Dahl D, et al. A transcriptional metastatic signature predicts survival in clear cell renal cell carcinoma. Nat Commun. 2022;13:5747. doi: 10.1038/s41467-022-33375-w 36180422 PMC9525645

[44] Chevrier S, Levine JH, Zanotelli VRT, Silina K, Schulz D, Bacac M, et al. An immune atlas of clear cell renal cell carcinoma. Cell. 2017;169:736–49.e18. doi: 10.1016/j.cell.2017.04.016 28475899 PMC5422211

[45] Bi K, He MX, Bakouny Z, Kanodia A, Napolitano S, Wu J, et al. Tumor and immune reprogramming during immunotherapy in advanced renal cell carcinoma. Cancer Cell. 2021;39:649–61.e5. doi: 10.1016/j.ccell.2021.02.015 33711272 PMC8115394

[46] Krishna C, DiNatale RG, Kuo F, Srivastava RM, Vuong L, Chowell D, et al. Single-cell sequencing links multiregional immune landscapes and tissue-resident T cells in ccRCC to tumor topology and therapy efficacy. Cancer Cell. 2021;39:662–77.e6. doi: 10.1016/j.ccell.2021.03.007 33861994 PMC8268947

[47] Hu J, Chen Z, Bao L, Zhou L, Hou Y, Liu L, et al. Single-cell transcriptome analysis reveals intratumoral heterogeneity in ccRCC, which results in different clinical outcomes. Mol Ther. 2020;28:1658–72. doi: 10.1016/j.ymthe.2020.04.023 32396851 PMC7335756

[48] Molgora M, Esaulova E, Vermi W, Hou J, Chen Y, Luo J, et al. TREM2 modulation remodels the tumor myeloid landscape enhancing anti-PD-1 immunotherapy. Cell. 2020;182:886–900.e17. doi: 10.1016/j.cell.2020.07.013 32783918 PMC7485282

[49] Wu TD, Madireddi S, de Almeida PE, Banchereau R, Chen YJ, Chitre AS, et al. Peripheral T cell expansion predicts tumour infiltration and clinical response. Nature. 2020;579:274–8. doi: 10.1038/s41586-020-2056-8 32103181

[50] Borcherding N, Vishwakarma A, Voigt AP, Bellizzi A, Kaplan J, Nepple K, et al. Mapping the immune environment in clear cell renal carcinoma by single-cell genomics. Commun Biol. 2021;4:122. doi: 10.1038/s42003-020-01625-6 33504936 PMC7840906

[51] Sjoberg E, Frodin M, Lovrot J, Mezheyeuski A, Johansson M, Harmenberg U, et al. A minority-group of renal cell cancer patients with high infiltration of CD20+B-cells is associated with poor prognosis. Br J Cancer. 2018;119:840–6. doi: 10.1038/s41416-018-0266-8 30293996 PMC6189087

[52] Helmink BA, Reddy SM, Gao J, Zhang S, Basar R, Thakur R, et al. B cells and tertiary lymphoid structures promote immunotherapy response. Nature. 2020;577:549–55. doi: 10.1038/s41586-019-1922-8 31942075 PMC8762581

[53] Brooks SA, Brannon AR, Parker JS, Fisher JC, Sen O, Kattan MW, et al. ClearCode34: a prognostic risk predictor for localized clear cell renal cell carcinoma. Eur Urol. 2014;66:77–84. doi: 10.1016/j.eururo.2014.02.035 24613583 PMC4058355

[54] Brannon AR, Reddy A, Seiler M, Arreola A, Moore DT, Pruthi RS, et al. Molecular stratification of clear cell renal cell carcinoma by consensus clustering reveals distinct subtypes and survival patterns. Genes Cancer. 2010;1:152–63. doi: 10.1177/1947601909359929 20871783 PMC2943630

[55] Rini B, Goddard A, Knezevic D, Maddala T, Zhou M, Aydin H, et al. A 16-gene assay to predict recurrence after surgery in localised renal cell carcinoma: development and validation studies. Lancet Oncol. 2015;16:676–85. doi: 10.1016/S1470-2045(15)70167-1 25979595

[56] Beuselinck B, Job S, Becht E, Karadimou A, Verkarre V, Couchy G, et al. Molecular subtypes of clear cell renal cell carcinoma are associated with sunitinib response in the metastatic setting. Clin Cancer Res. 2015;21:1329–39. doi: 10.1158/1078-0432.CCR-14-1128 25583177

[57] Giraldo NA, Becht E, Vano Y, Petitprez F, Lacroix L, Validire P, et al. Tumor-infiltrating and peripheral blood T-cell immunophenotypes predict early relapse in localized clear cell renal cell carcinoma. Clin Cancer Res. 2017;23:4416–28. doi: 10.1158/1078-0432.CCR-16-2848 28213366

[58] Clark DJ, Dhanasekaran SM, Petralia F, Pan J, Song X, Hu Y, et al. Integrated proteogenomic characterization of clear cell renal cell carcinoma. Cell. 2019;179:964–83.e31. doi: 10.1158/1538-7445.SABCS18-2458 31675502 PMC7331093

[59] Motzer RJ, Banchereau R, Hamidi H, Powles T, McDermott D, Atkins MB, et al. Molecular subsets in renal cancer determine outcome to checkpoint and angiogenesis blockade. Cancer Cell. 2020;38:803–17.e4. doi: 10.1016/j.ccell.2020.10.011 33157048 PMC8436590

[60] Wuttig D, Zastrow S, Fussel S, Toma MI, Meinhardt M, Kalman K, et al. CD31, EDNRB and TSPAN7 are promising prognostic markers in clear-cell renal cell carcinoma revealed by genome-wide expression analyses of primary tumors and metastases. Int J Cancer. 2012;131:E693–704. doi: 10.1002/ijc.27419 22213152

[61] Yao X, Qian CN, Zhang ZF, Tan MH, Kort EJ, Yang XJ, et al. Two distinct types of blood vessels in clear cell renal cell carcinoma have contrasting prognostic implications. Clin Cancer Res. 2007;13:161–9. doi: 10.1158/1078-0432.CCR-06-0774 17200351

[62] Denize T, Farah S, Cimadamore A, Flaifel A, Walton E, Sticco-Ivins MA, et al. Biomarkers of angiogenesis and clinical outcomes to cabozantinib and everolimus in patients with metastatic renal cell carcinoma from the phase III METEOR trial. Clin Cancer Res. 2022;28:748–55. doi: 10.1158/1078-0432.CCR-21-3088 34921022 PMC8866215

[63] Jilaveanu LB, Puligandla M, Weiss SA, Wang XV, Zito C, Flaherty KT, et al. Tumor microvessel density as a prognostic marker in high-risk renal cell carcinoma patients treated on ECOG-ACRIN E2805. Clin Cancer Res. 2018;24:217–23. doi: 10.1158/1078-0432.CCR-17-1555 29066509 PMC5904512

[64] Haas NB, Manola J, Uzzo RG, Flaherty KT, Wood CG, Kane C, et al. Adjuvant sunitinib or sorafenib for high-risk, non-metastatic renal-cell carcinoma (ECOG-ACRIN E2805): a double-blind, placebo-controlled, randomised, phase 3 trial. Lancet. 2016;387:2008–16. doi: 10.1016/S0140-6736(16)00559-6 26969090 PMC4878938

[65] Motzer RJ, Robbins PB, Powles T, Albiges L, Haanen JB, Larkin J, et al. Avelumab plus axitinib versus sunitinib in advanced renal cell carcinoma: biomarker analysis of the phase 3 JAVELIN renal 101 trial. Nat Med. 2020;26:1733–41. doi: 10.1038/s41591-020-1044-8 32895571 PMC8493486

[66] Wragg JW, Finnity JP, Anderson JA, Ferguson HJ, Porfiri E, Bhatt RI, et al. MCAM and LAMA4 are highly enriched in tumor blood vessels of renal cell carcinoma and predict patient outcome. Cancer Res. 2016;76:2314–26. doi: 10.1158/0008-5472.CAN-15-1364 26921326 PMC4875769

[67] Masiero M, Simoes FC, Han HD, Snell C, Peterkin T, Bridges E, et al. A core human primary tumor angiogenesis signature identifies the endothelial orphan receptor ELTD1 as a key regulator of angiogenesis. Cancer Cell. 2013;24:229–41. doi: 10.1016/j.ccr.2013.06.004 23871637 PMC3743050

[68] Niinivirta M, Georganaki M, Enblad G, Lindskog C, Dimberg A, Ullenhag GJ. Tumor endothelial ELTD1 as a predictive marker for treatment of renal cancer patients with sunitinib. BMC Cancer. 2020;20:339. doi: 10.1186/s12885-020-06770-z 32321460 PMC7179003

[69] Rautiola J, Lampinen A, Mirtti T, Ristimaki A, Joensuu H, Bono P, et al. Association of angiopoietin-2 and Ki-67 expression with vascular density and sunitinib response in metastatic renal cell carcinoma. PLoS One. 2016;11:e0153745. doi: 10.1371/journal.pone.0153745 27100185 PMC4839598

[70] Seeber A, Klinglmair G, Fritz J, Steinkohl F, Zimmer KC, Aigner F, et al. High IDO-1 expression in tumor endothelial cells is associated with response to immunotherapy in metastatic renal cell carcinoma. Cancer Sci. 2018;109:1583–91. doi: 10.1111/cas.13560 29498788 PMC5980224

